# Comprehensive review of the cervical ligamenta flava

**DOI:** 10.1007/s00276-025-03615-x

**Published:** 2025-04-01

**Authors:** Tatjana Mortell, Ali Mortezaei, Rarinthorn Samrid, Sassan Keshavarzi, Seiichi Inoue, Keishiro Kikuchi, Joe Iwanaga, Aaron S. Dumont, R. Shane Tubbs

**Affiliations:** 1 Tulane University School of Medicine, New Orleans, LA USA; 2 Department of Neurosurgery, Tulane Center for Clinical Neurosciences, Tulane University School of Medicine, New Orleans, LA USA; 3 Department of Neurology, Tulane Center for Clinical Neurosciences, Tulane University School of Medicine, New Orleans, LA USA; 4 Department of Anatomical Sciences, St. George’s University, St. George’s, Grenada; 5 Department of Structural and Cellular Biology, Tulane University School of Medicine, New Orleans, LA USA; 6 Department of Surgery, Tulane University School of Medicine, New Orleans, LA USA; 7 Department of Neurosurgery and Ochsner Neuroscience Institute, Ochsner Health System, New Orleans, LA USA; 8 University of Queensland, Brisbane, Australia; 9 Gonabad University of Medical Sciences, Gonabad, Iran; 10 Department of Orthopaedic Surgery, Kurume University, Kurume, Fukuoka Japan; 11 Division of Gross and Clinical Anatomy, Department of Anatomy, Kurume University School of Medicine, Kurume, Fukuoka Japan; 12 Department of Anatomy, Faculty of Medicine, Khon Kaen University, Khon Kaen, 40002 Thailand

**Keywords:** Anatomy, Cadaver, Review, Cervical spine, Ligament

## Abstract

**Purpose:**

The current literature contains many data associated with the cervical ligamentum flavum (CLF). The present study is to overview knowledge of CLF.

**Methods:**

Comprehensive literature review was performed.

**Results:**

Topics include anatomy, embryology, histology, radiology, clinical relevance, and pathological manifestations of the CLF, including ossification, calcification, and hypertrophy. Spine procedures always require extreme precision; spine surgeons and neurosurgeons encounter challenges that put patients’ lives at risk.

**Conclusion:**

This study can assist clinicians in performing spinal interventions with the fewest possible complications. Because there have been few studies of the CLF, further investigation is suggested.

## Introduction

The ligamentum flavum (LF), also known as the yellow ligament, is a posterior structure in the spinal canal essential for maintaining the stability and mobility of the spine and for attaching the laminae of the adjacent second cervical (C2) vertebra through the first sacral vertebral segment (S1), although some authors suggest it may extend from C1 [9, 15, 32]. Histologically, the LF comprises elastin and fibrillin fibers in each region. These fibers confer a distinct yellow color on the LF, making it more readily detectable during surgery [9, 15]. In 1938, Naffzinger et al. proposed an anatomical description of the LF. They described it as attaching to the inferior and anteroinferior surfaces of the cephalad portion of the lamina and penetrating the superior and posterosuperior surfaces of the caudal lamina [32].

**Fig. 1:**
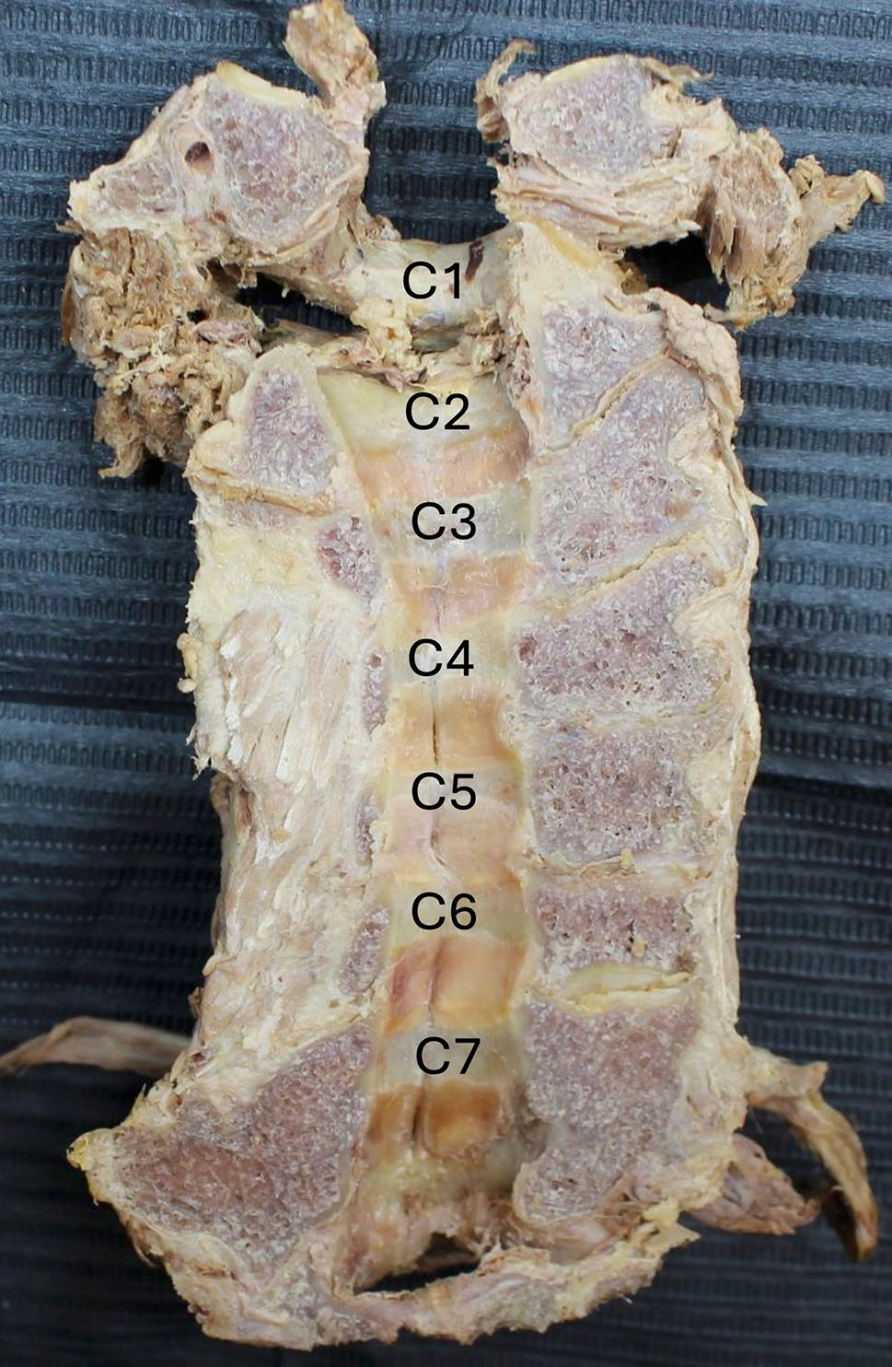
Anterior view of the posterior half of the cervical spine. Note that the ligamentum flavum becomes longer because of the increment in the interval between adjacent laminae

The LF is most commonly divided into three regions: cervical, thoracic, and lumbar. Owing to its location in the spinal canal, some authors have proposed a less common division: pars interspinalis, pars interlaminar, and pars capsularis [13, 19, 49]. Moving from C1 to C7, the LF becomes longer because of the increment in the interval between adjacent laminae (Fig. 1). It becomes thicker and narrower inferiorly in the spine, the lumbar LF being thickest. Notably, on imaging, the LF is thicker in spinal extension than in spinal flexion. Pathological changes such as hypertrophy, calcification, and ossification can affect its thickness [12, 28, 36, 49]. The most common LF pathology is hypertrophy [36].

LF is significant in spine surgery and epidural/subarachnoid anesthesia because of the frequency of such procedures, the sensitivity of the spine, and its position in the spinal canal. The cervical region is the second most common area for epidural steroid injection, a frequently conducted interventional procedure in the United States [1]. These examples highlight the need to recognize the anatomy of each portion of the LF accurately in surgical procedures such as microdiscectomy, cervical laminoplasty, and many others.

The cervical ligamentum flavum (CLF) occupies a more distinct and sensitive position than other parts of the LF. The slightest pathological alteration in it can cause relatively more severe and even irreversible complications. The CLF and its position should, therefore, always be considered by surgeons, anesthesiologists, and interventional specialists to ensure sufficient control and understanding. The present study is an opportunity to overview knowledge of CLF.

## Materials and methods

“A literature search was conducted using PubMed and Google Scholar with the keywords ‘ligamentum flavum,’ ‘ligamenta flava,’ and ‘yellow ligament.’ Abstracts were screened, and the full texts of relevant articles were selected, downloaded, and reviewed. ”

## Results

### Anatomy

#### The shape of the cervical ligamentum flavum

The CLF are paired ligaments (right and left) believed to attach the laminae of the C2 (or C1) to C7 vertebrae and cross in the midline, providing an ambiguous angle that is open anteriorly. Caudally, the LF thickens and becomes more prismatic, and its length increases as the laminae become larger, the distance between them widening sequentially [10, 53]. Sometimes, the right and left LF are not fused in the midline. In these cases, incomplete or complete gaps can form between them. The literature suggests that the incidence of such gaps ranges from 50 to 100%, with a mean of 74% in some studies. This also happens more frequently at the cervical and thoracic levels than the lumbar level [25–27, 61]. Nevertheless, various studies have described the LF as a structure with no midline separation [32, 38, 62]. The gap can vary and present as a fissure or foramen [26, 61].

#### Height

The LF and lamina height are identical and coincide, gradually increasing from cranial to caudal at subaxial cervical levels (C2–C7) [40, 42]. Males tend to have a higher LF than females, indicating that LF height is sex-dependent [3].

#### Width

The width of the CLF (distance between the midline and its lateral attachment) roughly follows the width of the laminae, which is relatively constant from C3–C4 to C7–T1 and narrowest at C2–C3 [40, 42].

#### Thickness

The LF is thinnest between the atlas (C1) and the axis (C2), so thin that it does not even appear in some people [10]; instead, it appears in the cervical region below, at C2–T1. The thickness of the LF is roughly constant except at the C2–C3 level, where it is markedly thinner than at other levels [9]. It also thickens from the lateral to the medial part of the ligament, leading to more muscular, bony attachments in the lamina's middle portion than the thinner lateral portion [56]. At C3 to C7, Sayit et al. revealed that the LF is thicker in spinal extension than flexion at the same level. In contrast, LF thickness did not change appreciably at the C2–C3 and C7–T1 levels in extension, flexion, or neutral positions [49].

According to Kim et al., the mean cervical LF thicknesses in a control group and a cervical spinal stenosis group were 1.48 mm and 2.09 mm, respectively (p < 0.01) [22].

#### Attachments, neural foramen, and laminar coverage

The CLF does not quite reach the neural foramen, and its lateral margins are roughly settled in important points in that foramen. The LF also encompasses the anterior side of the cervical facet joint and the craniomedial side of the joint corner [3, 56]. The CLF mainly attaches to the anterior cranial surface of the caudal lamina and does not adhere to the posterosuperior part of the lamina [61]. Unlike laminar coverage, the empty zone steadily shrinks from cranial to caudal in the cervical levels [40, 56]. CLF laminar coverage steadily rises from 33% in the para midline at the C2 level to 70% in the para midline at the C6 level [40]. The mean cervical LF areas in the control and cervical spinal stenosis groups are 25.24 mm^2^ and 45.34 mm^2^, respectively (p < 0.01) [22].

### Embryology

Current literature suggests that the LF is a paired structure, and its development is strongly associated with laminae. In some investigations, LFs appeared at 10–11 weeks [30], but Misawa et al. recognized ossification centers of laminae and LFs only at 12 weeks gestational age [29]. At 15 weeks of development, the LF becomes thicker, although it does not stain as strongly as an adult LF [29, 30].

### Histology

The LF is an orange-yellow ligament, or pink in some cases [15, 40]. It comprises 80% elastic and 20% collagen fibers [44, 60]. The various sizes of elastic fibers intermingle with the collagen fibers in a predominantly parallel fashion, but some fibers cross over to form an arched arrangement [11, 56]. Notably, in some parts of the attachment area of the ligament, there are diamond-shaped interstices between the elastic and collagen fibers, and collagen fibers prevail in this area. Further, this region has fewer and smaller elastic fibers, with a modest number of fibroblasts scattered among them [11, 43]. Occasionally, there are vessels among the collagen fibers, most commonly at the inner border of the ligament, the outer border often encircled by muscle fibers [43]. The attachment point of the ligament to the vertebral lamina is occasionally characterized by fibrous tissue and bone adjacent to the edge of the ligament cartilaginous tissue [43, 51].

### Radiology

Imaging modalities such as magnetic resonance (MRI), computed tomography (CT), and ultrasound can assess CLF pathologies such as ossification, hypertrophy, and calcification. CT and MRI are both useful for diagnosing CLF pathologies. However, MRI is recommended for diagnosing such pathologies given its greater soft-tissue contrast resolution, multiplanar capabilities, absence of beam-hardening artifacts, and ionizing radiation. However, a CT scan can be an excellent surrogate for MRI owing to its availability and the more significant economic constraints of MRI imaging, among its additional benefits. Another potential advantage of CT is that it is better for detecting bone anomalies relevant to ligament injuries [45, 50]. CT scans are also widely available to assess cervical vertebra fracture patterns in acute situations. A high-quality CT scan with appropriate interpretation can identify fracture patterns, avulsion fractures, and concurrent ligament injuries in the cervical spine [57]. However, a cervical spine MRI is the most effective and sensitive imaging modality for evaluating the cervical vertebrae and their components, such as the cervical facet, LF, and intervertebral disc. It is also superior in demonstrating the structures of soft tissues, such as the intervertebral disc and LF [55, 63]. In one study, the CT and MRI measurements were practically identical, and just one of those imaging modalities was adequate for measuring the LF and other spinal structures accurately. The authors of this study also found that the LF appeared slightly thicker on CT than on MRI, and on average, it appeared smaller on T1-weighted sequences than on T2-weighted sequences [50]. Kinetic MRI (fMRI) is another imaging modality that could be more efficient for elucidating the pathogenesis of spinal canal stenosis in flexion and extension [63].

Ultrasound imaging is another modality referenced in the literature. It is suggested to be superior in providing approximate results most quickly. It is also a less invasive, more mobile, and easier to handle modality than CT or MRI imaging. One suggested use of ultrasound is clearly identifying the epidural space [6, 21, 39]. The literature indicates that the most accurate method for using ultrasound imaging of the LF is to measure the distance from the skin to the inner surface of the LF [6]. However, Kim et al. demonstrated that ultrasound imaging shows less actual needle depth [21].

### Clinical relevance

#### Ossification

Ossification of the LF is a slowly progressing disorder whose etiology is still unclear. It is most often detected after the onset of symptoms associated with spinal cord compression. Plain cervical spine radiography can be used for screening [18]. Ossification of the LF is not a rare disorder; its estimated prevalence is 4.3–26% [14, 59]. Guo and colleagues [14] reported the exam findings of 1736 participants. They found prevalences of 4.3% in cervical ossification and 0.1% in lumbar ossification of the LF. The CLF ossification sites affected ranged from the middle to the lower cervical spine. In order of significance, genetic predisposition, mechanical stress, obesity, reduced parathormone levels, and overexposure to fluoride are the main etiological factors hypothesized to be responsible for LF ossification [23, 31, 52]. The most active ossification process is the articular surface of the intervertebral joints and the point where the fistula of the joint connects to the LF [17].

The symptoms of CLF ossification vary depending on the level of the spine where the pathological changes arise. They are similar to the symptoms of hypertrophy and calcification but differ in severity. Studies indicate that they include loss of dexterity in the hands, especially fine movements such as writing or removing coins from pockets, upper and lower limb paresthesiae, numbness, muscular weakness and stiffness, and pain associated with radiculopathy, sometimes in the neck [24]. Urinary symptoms usually manifest as urgency, but there can also be bladder and bowel incontinence [23]. The neurological manifestations of CLF ossification can be greater when the cervical spine rather than the lumbar and thoracic spine is affected [23, 52]. Ossification of LF is diagnosed based on clinical symptoms, lateral X-rays, CT scans, and MRIs.

#### Hypertrophy

LF hypertrophy was initially identified in 1913 as a primary cause of spinal stenosis, which can lead to systemic disorders, limb pain, paresthesiae, and paralysis [46, 65]. The terms LF thickening or hypertrophy are used interchangeably in the literature to describe LF buckling related to disc degeneration and successive decreases in disc height. Whether LF hypertrophy is related to enhanced LF thickness or reduced disc height leading to LF buckling into the spinal canal is unclear [4, 48]. According to preliminary studies, a range of molecular and genomic factors, such as genome-wide DNA methylation, cytokine receptor-like factor 1, and microRNAs, are implicated in LF hypertrophy [8, 64]. LF flexibility declines in the elderly, but the exact mechanism for this decline is unknown. A few publications indicate that aging causes ligament fibrosis and an increase in the collagen-to-elastin ratio, thus reducing the elasticity of the LF. Other publications presume that the decrease in flexibility is due merely to buckling of the ligament owing to disc degeneration, reduction in disc height, and an eventual reduction in spinal canal diameter leading to spinal canal stenosis [37, 47, 54]. There are no sex differences in LF hypertrophy, unlike disc degeneration. In addition, the right side of the LF thickens more than the left [54].

#### Calcification

**Fig. 2:**
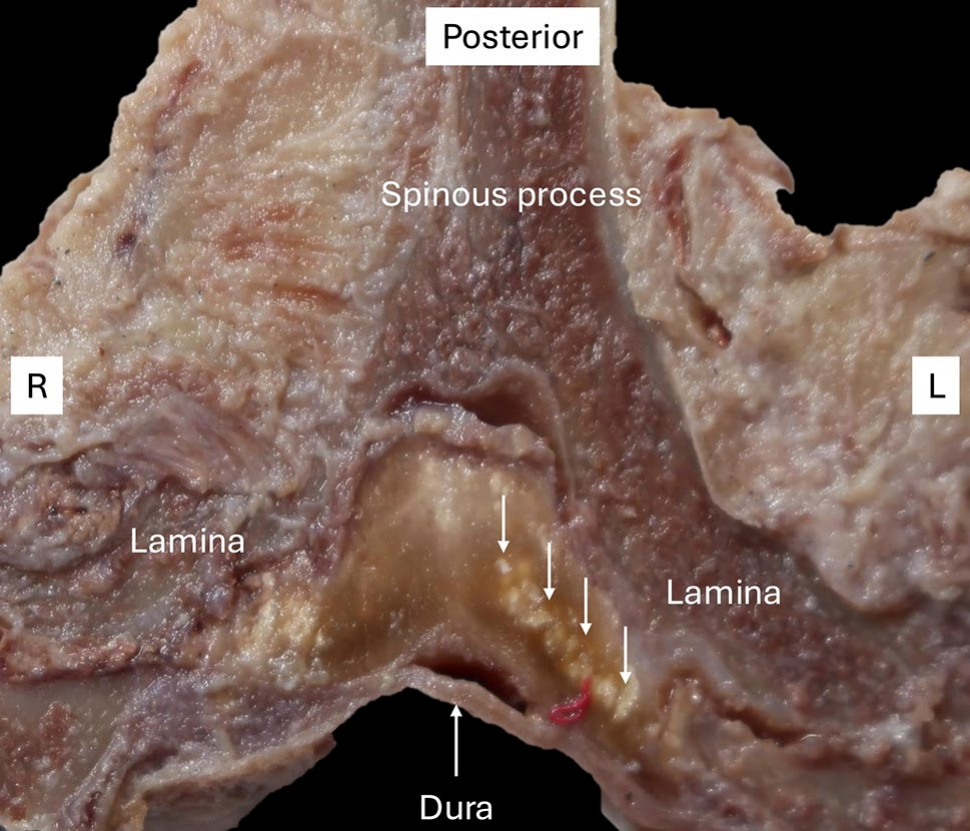
Superior view (axial section) of the calcified ligamentum flavum (arrows) in the cervical spine

Nanko and colleagues identified calcification of LF for the first time in 1976 [34]. This crystal deposition disease mainly affects the central portion of the LF (Fig. 2). Its pathogenesis has been described in relation to degenerated and thickened ligaments. Calcium deposits mainly occur in the central part of the LF, which is surrounded by degenerated elastic fibers. Typically, calcification of the LF has no continuity with the lamina, and the superficial and deep layers of the LF are preserved [33, 35].

Many confounding conditions can affect the development of LF calcification. These include aging, endocrine imbalance, metabolic diseases, cervical spine mechanical stress, and chondrocyte metaplasia [35, 41]. In one study, researchers found that 85% of all patients with LF calcification were female and had an average age of 64.8 years (39–80 years). Most of the lesions in this population were found between the C4-5 and C6-7 levels. This study provided some information, but the prevalence of LF calcification in the general population needs to be clarified [7]. Additionally, in a radiological study, 0.09% of patients complaining of neck problems had radiological findings indicative of LF calcification [58].

According to the literature, LF calcification mainly affects Asian populations and patients older than 60, mostly females. It is sometimes associated with cervical disc disease. Concomitant calcium deposits have also been observed in other articular or periarticular sites, such as the knee joint, intervertebral discs, hip joint, pubic symphysis, and shoulder [55, 63]. Gait disturbances coexist with an abnormal deep tendon reflex or sensory disturbance. Sensory disturbance and hand weakness are also common [7, 55].

## Discussion

The CLF is the posterior entrance gateway to the cervical spinal cord. Interventions on it are performed in millions of cases of epidural analgesia, interlaminar epidural steroid injections, and microdiscectomy. Because of its sensitivity, accurate procedures are needed to prevent further complications, and it is critical in these circumstances to understand the anatomy of the CLF exactly.

The LF is a protective structure that supports the posterior section of the spine and is one of the most important structures in epidural anesthesia. The cervical portion of the LF is significant in any intervention because of its location. The LF develops at about 12 weeks of gestation [29] and is composed of elastic and collagen fibers [44], which give it its characteristic yellow appearance [15]. Anatomically, from cranial to caudal, the CLF becomes larger, thicker, and prism-like [10, 40, 42, 53]. Inferiorly, its height and length increase gradually, being greater at the C7–T1 levels than at other levels [40, 42, 53]. Since the width of the CLF is proportional to the width of the lamina, it is mostly constant, but it is narrower at the C2–C3 level than at other levels [40, 42]. Gaps in the LF occur with an approximate mean incidence of 75% [26, 27], making it a non-integrated structure. Therefore, one cannot always rely on the CLF as a perceptible barrier to epidural needle placement at cervical levels. CLF non-integration can also explain the procedure's absence or a false sense of “loss of resistance” [19, 25–27]. However, some investigations have reported no gaps in the LF [32, 38, 53].

Since the LF is located immediately adjacent to the nerve components of the spinal cord, any pathology that involves the slightest change in it could cause neurological disturbances. The reported pathologies of the CLF include ossification, hypertrophy, and calcification. In general, all these disorders have identical symptoms, such as muscular weakness and stiffness, gait disturbance, numbness, limb paresthesia or plegia, and pain. Symptoms vary depending on the location of the spinal cord involvement, but ossification usually produces more severe symptoms than the other pathologies. It mainly involves the thoracic part of the LF rather than the cervical and lumbar parts [14, 59]. One explanation for the high frequency of LF ossification at the upper and lower thoracic levels is increased mechanical stress, where the thoracic vertebrae form the junction between the stiff rib cage and the cervical or lumbar spine [36]. In patients with LF ossification, alterations are prevalent in other spinal structures, such as the vertebral ligaments, vertebral body, intervertebral disc, facet joint, and paravertebral muscles [16].

The CLF is associated with spinal decompression surgeries, particularly for treating spinal stenosis, myelopathy, and radiculopathy [33, 34]. Procedures such as laminectomy, laminoplasty, foraminotomy, and posterior cervical decompression surgery may be performed. Diagnosis of an LF pathology is based on neurological findings, examination, and imaging modalities using MRI, CT, and ultrasound [6, 21, 39, 45, 50]. At the cervical and lumbar levels, the pathological level can be determined by neurological and anatomical findings of the severity of the muscular lesion, sensory and gait disturbance, and the degree of deep tendon reflex [2, 35, 41, 65]. Thoracic myelopathy is suspected in individuals with lower deep tendon reflexes in the lower than the upper limb. In these cases, imaging investigations from the upper to lower thoracic levels are required [16]. Wide imaging investigations are needed in patients with flaccid paralysis of the lower limbs, especially those with substantial muscular atrophy and weakness, since problems at the lower thoracic level, such as LF abnormalities and/or posterior longitudinal ligament disturbances, are suspected [5]. MRI is a safer and more convenient imaging modality for the best imaging of soft tissues, but a CT scan can help in an emergency [50, 51]. However, there are no consistent correlations between the degree of compression and the severity of neurological findings in pathologies that compress the spinal cord. Some individuals have minimal or no neurological signs, even when the spinal cord is severely compressed [20]. This is a crucial point to consider before making a diagnosis.

## Conclusion

Spine procedures always require extreme precision, so spine surgeons and neurosurgeons encounter challenges that put patients’ lives at risk. This study can assist clinicians in performing spinal interventions with the fewest possible complications. Because there have been few studies on the CLF, further investigation is suggested.

## Data Availability

No datasets were generated or analysed during the current study.
